# Microbial interactions in the mosquito gut determine *Serratia* colonization and blood-feeding propensity

**DOI:** 10.1038/s41396-020-00763-3

**Published:** 2020-09-07

**Authors:** Elena V. Kozlova, Shivanand Hegde, Christopher M. Roundy, George Golovko, Miguel A. Saldaña, Charles E. Hart, Enyia R. Anderson, Emily A. Hornett, Kamil Khanipov, Vsevolod L. Popov, Maria Pimenova, Yiyang Zhou, Yuriy Fovanov, Scott C. Weaver, Andrew L. Routh, Eva Heinz, Grant L. Hughes

**Affiliations:** 1 Department of Pathology, University of Texas Medical Branch, Galveston, TX USA; 2 Departments of Vector Biology and Tropical Disease Biology, Centre for Neglected Tropical Disease, Liverpool School of Tropical Medicine, Liverpool, UK; 3 World Reference Center for Emerging Viruses and Arboviruses, Institute for Human Infections and Immunity, and Department of Microbiology and Immunology, University of Texas Medical Branch, Galveston, TX USA; 4 Department of Pharmacology and Toxicology, Sealy Center for Structural Biology, University of Texas Medical Branch, Galveston, TX USA; 5 Department of Paediatrics and Tropical Medicine, Baylor College of Medicine, Houston, TX USA; 6 The Institute for Translational Science, University of Texas Medical Branch, Galveston, TX USA; 7 Institute for Global Health and Translational Science and SUNY Center for Environmental Health and Medicine, SUNY Upstate Medical University, Syracuse, NY USA; 8 Institute of Integrative Biology, University of Liverpool, Liverpool, UK; 9 Department of Biochemistry and Molecular Biology, University of Texas Medical Branch, Galveston, TX USA; 10 Departments of Vector Biology and Clinical Sciences, Liverpool School of Tropical Medicine, Liverpool, UK

**Keywords:** Symbiosis, Microbial ecology, Bacterial host response

## Abstract

How microbe–microbe interactions dictate microbial complexity in the mosquito gut is unclear. Previously we found that, *Serratia*, a gut symbiont that alters vector competence and is being considered for vector control, poorly colonized *Aedes aegypti* yet was abundant in *Culex quinquefasciatus* reared under identical conditions. To investigate the incompatibility between *Serratia* and *Ae. aegypti*, we characterized two distinct strains of *Serratia marcescens* from *Cx. quinquefasciatus* and examined their ability to infect *Ae. aegypti*. Both *Serratia* strains poorly infected *Ae. aegypti*, but when microbiome homeostasis was disrupted, the prevalence and titers of *Serratia* were similar to the infection in its native host. Examination of multiple genetically diverse *Ae. aegypti* lines found microbial interference to *S. marcescens* was commonplace, however, one line of *Ae. aegypti* was susceptible to infection. Microbiome analysis of resistant and susceptible lines indicated an inverse correlation between *Enterobacteriaceae* bacteria and *Serratia*, and experimental co-infections in a gnotobiotic system recapitulated the interference phenotype. Furthermore, we observed an effect on host behavior; *Serratia* exposure to *Ae. aegypti* disrupted their feeding behavior, and this phenotype was also reliant on interactions with their native microbiota. Our work highlights the complexity of host–microbe interactions and provides evidence that microbial interactions influence mosquito behavior.

## Introduction

Mosquitoes harbor a variety of diverse microbes that profoundly alter host phenotypes [1–3]. In general, the bacterial microbiome can vary considerably between mosquito species and individuals, but within an individual, it comprised relatively few bacterial taxa [4, 5]. It is becoming more apparent that a variety of factors contribute to this variation, but we have a lack of understanding regarding why some taxa are present in a host, yet others are absent. In mosquitoes and other insects, much effort has been undertaken to characterize the infection status of species and populations for specific endosymbiotic bacteria such as *Wolbachia* [6–9], yet few studies have examined the infection prevalence of specific gut-associated bacteria in mosquito vectors. It is evident that several gut-associated bacterial taxa are common between phylogenetically diverse mosquito species [4, 5], but less attention has been paid to identifying incompatible hos–microbe associations and the mechanism(s) behind this incompatibility.

Microbiome assembly in mosquitoes is influenced by the environment, host and bacterial genetics, and stochastic processes. While the host is instrumental in maintaining microbiome homeostasis [10–14], evidence is emerging that bacterial genetics and microbe–microbe interactions also dictate the prevalence and abundance of microbiota [15–18]. These processes are important as the microbiome can influence the ability of mosquitoes to transmit pathogens [3, 19, 20], but potentially other traits related to vectorial capacity. Evidence from other insect systems shows a role for commensal microbes influencing behavior [21, 22]. In mosquiotes, pathogen infection can also reduce feeding rates and host seeking behavior, possibly by altering expression of odorant binding proteins or immunity-related genes [23–26]; however, less know how gut-associated microbes alter these phenotypes. Therefore, a greater appreciation of factors that influence colonization of the mosquito gut and how microbes effect the host is critical for deploying microbial-based approaches to control mosquito-borne disease [23, 24].

*Serratia* is a ubiquitous genus of gut symbionts that is known to infect a diverse array of insects, including taxa within the Homopteran, Hymenopteran, Dipteran, and Lepidopteran orders [25–30]. Several medically relevant vectors also harbor this bacterium [31–35]. In mosquitoes, *Serratia* appears to broadly infect Culicine and *Anopheles* mosquitoes [36–39], and these infections can have important phenotypic effects including altering the ability of these vectors to transmit pathogens [40–42]. Previosuly, we found a species-specific infection cline in *Serratia* levels in *Culex quinquefasciatus*, *Ae. albopictus,* and *Ae. aegypti* mosquitoes reared under identical conditions within the same insectary [4]. *Serratia* was a dominant member of the microbiota within *Cx. quinquefasciatus*, infected *Ae. albopictus* at low levels, and poorly infected or was absent from *Ae. aegypti* [4]. We also found that field-collected *Ae. aegypti* from the Houston region (USA) lacked *Serratia* [4].

Other studies have found variable results in respect to the prevalence of *Serratia* in the yellow fever mosquito. Using high-throughput 16S rRNA amplicon sequencing, *Serratia* was found to be absent or at low levels in some *Ae. aegypti* field populations [5, 40–43], yet present in others [37, 44]. Culture-dependent approaches have also confirmed the presence of *Serratia* in this mosquito species [45–48]. The variable nature of infection in the field could be due to the presence or absence of this bacterium in the local aquatic environment; however, this does not explain the infection cline we observed in our insectary when rearing *Ae. aegypti* given that *Cx. quinquefasciatus*, which reared in the same insectary, was heavily infected [4]. The lack of *Serratia* infection in these lab-reared *Ae. aegypti* mosquitoes suggests that there is a maladaptation between this particular mosquito line and *Serratia* strains.

To investigate the incompatibility between *Serratia* and the yellow fever mosquito, we isolated and characterized two distinct strains of *S. marcescens* present within the *Cx. quinquefasciatus* microbiome, and examined their ability to infect *Ae. aegypti*. We found that both *S. marcescens* strains poorly infected several *Ae. aegypti* lines. However, inducing dysbiosis in the native microbiota with antibiotics facilitated infections, suggesting the incompatibility was related to microbe–microbe interactions. In addition to microbial antagonism, we found that infection with these *S. marcescens* strains disrupted the feeding behavior of mosquitoes. We further show the phenotypes induced by *S. marcescens* are driven by interactions with *Enterobacteriaceae* bacteria. Our work highlights the complexity of host–microbe interactions and provides further evidence that microbial exclusion influences microbiome composition and abundance within mosquitoes. These results are also relevant in the context of the holobiont, whereby both the host and the associated microbiota dictate organism phenotypes.

## Methods

### Mosquito rearing

Colony mosquitoes were reared at 27 °C with 80% humidity in the UTMB insectary. Mosquitoes were fed 10% sucrose ad libitum and maintained at a 12:12 light:dark cycle. Mosquitoes were fed with defibrinated sheep blood (Colorado Serum Company, Denver, CO) using a hemotek membrane feeder. Table S1 lists the colony mosquitoes used in experiments.

### Isolation and characterization of *S. marcescens* from *Culex quinquefasciatus*

Homogenates of *Cx. quinquefasciatus* were stored in PBS at −80 °C as a glycerol stock. *S. marcescens* was isolated using conventional microbiological culturing. Briefly, LB plates were inoculated and incubated at 30 °C. Individual bacterial colonies were selected and purified from two different *Culex* mosquitoes. Two *S. marcescens* strains, named CxSm1 and CxSm2, were selected. Both strains had a red pigmentation, although intensity of the color varied between strains. In addition, there were differences in swimming motility and oxidase activity. These strains were subcultured for species identification by PCR amplifying the variable region of the 16S ribosomal RNA gene using universal bacterial primers. Primer sequences are listed in Table S2. Swimming motility was determined by inoculating LB medium (0.35% agar), incubating at 30 °C overnight, and then quantifying motility toward the periphery of the plate [49]. DB BBL^TM^ oxidase reagent droppers (BD & Comp., Sparks, MD) were used to detect cytochrome oxidase activity in bacteria following the manufacturer’s instructions. Scanning electron microscopy was conducted as previously described [50, 51].

### Selection of *S. marcescens* antibiotic-resistant mutants

*S. marcescens* antibiotic-resistant mutants were created as described [52] with some modification. Briefly, tubes containing 5 ml of LB broth with different concentrations of streptomycin (Sm) (Sigma) and rifampicin (Rif) (Sigma) (range: 0 [control] and 5, 10, 25, 50 μg/ml) were inoculated with 0.1 ml of a dilution of the bacterial cultures to obtain an inoculum of ~10^6^ colony forming unit (CFU)/ml. After overnight incubation at 30 °C, bacterial aliquots from the tubes with the highest concentration of appropriated antibiotic were inoculated in LB broth [Sm supplemented (range: 0 [control], 25, 50, 100 μg/ml) and Rif supplemented (range: 0 [control], 50, 100, 200, 250, 500 μg/ml)] and incubated overnight at 30 °C. Finally, after several passages in the presence of corresponding antibiotics, the CxSm1^RifR^ (MIC 400 μg/ml) and CxSm2^SmR^ (MIC 150 μg/ml) mutants were selected. The same approach was used to create a *Cedecea*^RifR^ mutant.

### Oral infection of mosquitoes with *S. marcescens*

The *S. marcescens* CxSm1^RifR^ and CxSm2^SmR^ strains were used for mosquito oral infection. Bacteria were grown in a 25-ml LB medium overnight culture at 30 °C containing either Rif (200 μg/ml) or Sm (100 μg/ml). Bacteria were pelleted by centrifugation at 5000 rpm for 20 min and then washed twice with sterile PBS and suspended in 2.5-ml PBS. Bacterial PBS stock was titrated by serial dilutions and quantified by plating on LB agar and measuring CFUs. The bacterial PBS stock dilutions were resuspended in 10% sterile sucrose to a final concentration of 1 × 10^7^ cells/ml. When supplementing antibiotics in the sugar meal, Rif (200 μg/ml) or Sm (100 μg/ml) was added to the sucrose solution. Mosquitoes were fed with a bacterial infected solution for 3 days. Then, mosquitoes were fed with 10% sterile sucrose or 10% sterile sucrose plus corresponded antibiotic, as required. At each time point, ten mosquitoes from each group were aspirated, surface sterilized, and homogenized in 250-μl PBS separately. Serial dilutions of mosquito homogenate were plated on LB agar and LB agar with the appropriate antibiotic and CFUs quantified. Experiments were repeated twice or three times as described in the figure legends.

### Microbiome analysis of *Ae. aegypti* lines

The microbiomes of *Ae. aegypti* lines were analyzed using barcoded high-throughput amplicon sequencing of the bacterial *16S rRNA* gene using a similar approach as previously described [4, 53]. DNA was extracted (QIAamp DNA Mini kit, QIAgen, Valencia, CA) from individual whole surface sterilized mosquitoes 5 days post eclosion (*N* = 15). To evaluative possible contamination, a spike in positive control [54] was amplified under the same conditions as genomic DNA isolated from mosquitoes. The spike in control was synthesized as a gBlock (Intergrated DNA Technologies, Coralville, IA) and 100 pmole of template was used as template for PCRs. High-throughput sequencing of the bacterial 16S ribosomal RNA gene was performed using gDNA isolated from each sample. Sequencing libraries for each isolate were generated using universal 16S rRNA V3-V4 region primers in accordance with Illumina 16S rRNA metagenomic sequencing library protocols [55]. The samples were barcoded for multiplexing using Nextera XT Index Kit v2 (Illumina, San Diego, CA). Sequencing was performed on an Illumina MiSeq instrument using a MiSeq Reagent Kit v2 (500-cycles). To identify the presence of known bacteria, sequences were analyzed using the CLC Genomics Workbench 11.0.1 Microbial Genomics Module. Reads were trimmed of sequencing adapters and barcodes, and any sequences containing nucleotides below the quality threshold of 0.05 (using the modified Richard Mott algorithm) and those with two or more unknown nucleotides or sequencing adapters were removed. Reference-based OTU picking was performed using the SILVA SSU v128 97% database [56]. Sequences present in more than one copy but not clustered to the database were placed into de novo OTUs (99% similarity) and aligned against the reference database with 80% similarity threshold to assign the “closest” taxonomical name where possible. Chimeras were removed from the dataset if the absolute crossover cost was three using a *k*-mer size of six. Alpha diversity was measured using Shannon entropy (OTU level), rarefaction sampling without replacement, and with 100,000 replicates at each point. Beta diversity was calculated and nonmetric multidimensional scaling (NMDS) plots were created using Bray–Curtis dissimilarity. Differentially abundant bacteria (family level) were identified using analysis of composition of microbiomes (ANCOM) with a significance level of *p* < 0.05 [57].

The total *Serratia* load within each mosquito line was assessed by qPCR. The S-adenosylhomocysteine nucleosidase (PFS) gene of *Serratia* was amplified with the primers psf1-F and psf-R [58]. The *Ae. aegypti* or *Cx. quinquesfactius S7* gene was amplified with aeg-S7-F and aeg-S7-R or Cq-S7-F and Cq-S7-R primers, respectively [4]. The PCR was done in a 10-μl reaction containing 1 μM of each primer, 1× SYBR Green (Applied Biosystems, Carlsbad, CA) and 2 μl of genomic DNA template. Cycling conditions involved an initial denaturation at 95 °C for 10 min, 40 cycles of 15 s at 95 °C, 1 min at 60 °C. Fluorescence readings were taken at 60 °C after each cycle before deriving a melting curve (60–95 °C) to confirm the identity of the PCR product. The PCR was carried out on the ABI StepOnePlus Real-Time PCR System. Relative abundance was calculated by comparing the *Serratia* load to the single-copy mosquito gene.

### Life history assays

To determine blood-feeding success, mosquitoes were offered a sheep blood meal using a hemotek feeding system. Cups of 25 female mosquitoes were starved for 24 h prior to blood feeding. Mosquitoes were given the opportunity to feed, and then the number of blood-fed mosquitoes were counted. For a subset of mosquitoes, the prevalence of *S. marcescens* in blood-fed and non-blood-fed mosquitoes was determined by plating on selective media. To examine the reproductive output, we measured the number of eggs produced by a blood feed female. Individual blood-fed females were placed into a vial with an oviposition site. After 4 days, the number of eggs were counted. Females that did not lay were excluded from the analysis. For most assays, the mortality of mosquitoes was quantified daily by counting and removing dead mosquitoes in cups.

### Genome sequencing

DNA isolation from bacteria was done using the PureLink™ Genomic DNA Mini Kit (Thermo Scientific, Carlsbad, CA). The Oxford Nanopore Technologies’s (ONT) MinION libraries were created with the 1D Native barcoding genomic DNA kit (with EXP-NBD103 and SQK-LSK108), following standard protocol (ver. NBE_9006_v103_revO_21Dec2016). In brief, 1.5 µg of each genomic DNA was fragmented (Covaris g-TUBE), end-repaired (NEBNext^®^ Ultra™ II End Repair/dA-Tailing Module, New England Biolabs, Ipswich, MA), barcodes are ligated, pooled in equal-molar amounts and finally adapter ligated. The pooled library was loaded to a FLO-MIN106 flow cell and sequenced using the default settings of the MinKNOW for at least 24 h. Base calling was conducted with *Albaco*re (release 2.3.3, https://nanoporetech.com/) with the following parameters: -k SQK-LSK108 -f FLO-MIN106 --barcoding. Data trimming and quality filtering was conducted with *Porechop* (https://github.com/rrwick/Porechop) with the following parameter: --discard_unassigned.

In addition, bacterial strains were submitted for short-read Illumina sequencing to 30X coverage using the Standard Whole-Genome Service from the MicrobesNG service (www.microbesng.uk, Birmingham, UK). Assemblies were performed using unicycler [59], generating a hybrid assembly using both long- and short-read sequences as input for each strain, respectively, for the assembly process. FastANI (average nucleotide identity) was used on a set of *Serratia* reference genomes retrieved from NCBI (Table S3) to confirm the species allocation. ANI analysis shows that CxSm1 and CxSm2 are highly similar to *Serratia* sp. Y25, which likely forms a subspecies of *S. marcescens* with an average ANI distance of 0.054 (Table S4 and Fig. S1); there was no difference in ANI level between CxSm1 and CxSm2. Mapping against *S. marcescens* reference strains thus resulted in high numbers of single nucleotide polymorphisms (SNPs; 252,113 and 253,191 for CxSm1 and CxSm2, respectively, against NZ_HG326223 DB11); whereas 44,435 and 44,913 SNPs were detected when mapping against the *Serratia* sp. YD25 genome (CP016948.1). 44,169 of these were ACGT-only sites where at least one of the sequences differs from the reference; 29 of these core genome SNPs differ between CxSm1 and CxSm2. Mapping was performed using SMALT v0.7.4 (ref: SMALT: a mapper for DNA sequencing reads. Available from: https://sourceforge.net/projects/smalt/) to produce a BAM file. Variation detection was performed using SAMtools mpileup v0.1.19 [60] with parameters “-d 1000 -DSugBf” and bcftools v0.1.19 (ref: bcftools: utilities for variant calling and manipulating VCFs and BCFs. Available from: http://samtools.github.io/bcftools/) to produce a BCF file of all variant sites. The option to call genotypes at variant sites was passed to the bcftools call. All bases were filtered to remove those with uncertainty in the base call. The bcftools variant quality score was required to be >50 (quality < 50) and mapping quality greater than 30 (map_quality < 30). If the same base call was not produced by all reads, the allele frequency, as calculated by bcftools, was required to be either 0 for bases called the same as the reference, or 1 for bases called as a SNP (af1 < 0.95). The majority base call was required to be present in at least 75% of reads mapping at the base (ratio < 0.75), and the minimum mapping depth required was four reads, at least two of which had to map to each strand (depth < 4, depth_strand < 2). Finally, strand_bias was required to be <0.001, map_bias <0.001, and tail_bias <0.001. If any of these filters were not met, the base was called as uncertain. All sequence data are available under BioProject number PRJNA641526.

## Results

### *Serratia* strain characterization

Two strains of *Serratia* were isolated from *Cx. quinquefasciatus* by conventional microbiology procedures. 16S rRNA sequencing indicated that these strains were *S. marcescens*, and each produced a red pigmentation when grown in a culture, which is indicative of this species (Fig. S2a). Although the 16S rRNA sequence was identical between strains, we saw phenotypic differences in their swimming motility, oxidase activity, and capacity to form biofilms, suggesting that they were phenotypically divergent (Fig. S2a, b). Swimming motility has been implicated in host gut colonization of several hosts [61, 62], and these traits can influence pathogen infection in mosquitoes [63]. To further characterize these strains (named CxSm1 and CxSm2), we sequenced their genomes using nanopore and Illumina technologies. Comparative genome analysis indicated high similarity between the two strains (no difference reported at ANI level; 29 core SNPs differed between CxSm1 and CxSm2 when compared to *Serratia* sp. Y25; for details see “Methods”). They showed 94.7% ANI similarity to *S. marcescens* when comparing with a set of *Serratia* reference genomes, indicating that these might represent a subspecies of *S. marcescens* (Fig. S1 and Tables S3, S4). Recent work has indicated a population structure in *S. marcescens* with at least two different clades [64], which might be an indication for several subspecies or indeed a species complex, as is for example seen for *Klebsiella pneumoniae* or *Enterobacter cloacae* [65, 66]. To aid our recovery of each of these *S. marcescens* strains on media, we selected for rifampicin and streptomycin spontaneous antibiotic-resistant isolates for CxSm1 and CxSm2, respectively (antibiotic-resistant strains named CxSm1^RifR^ and CxSm2^SmR^).

### *Serratia* colonization of mosquitoes

**Fig. 1:**
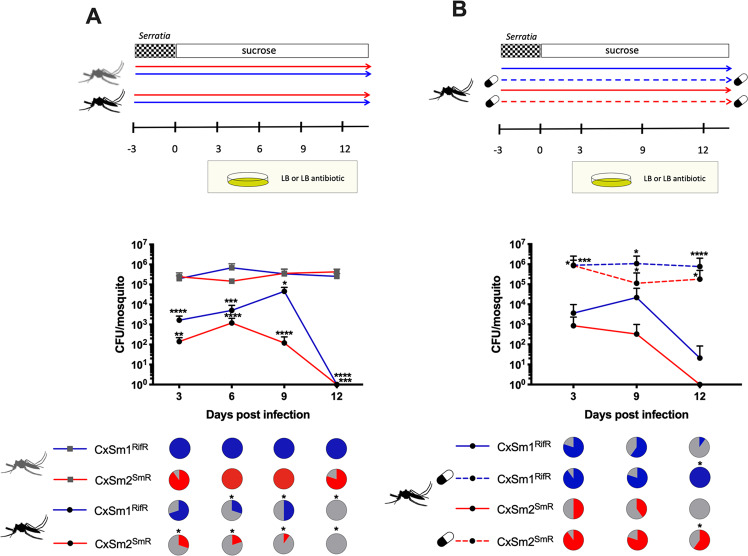
*Serratia* infections in native and non-native mosquito hosts. Infection of CxSm1^RifR^ and CxSm2^SmR^ into *Cx. quinquefasciatus* (gray) and *Ae. aegypti* (black) mosquitoes (**a**). Solid lines indicate rearing on sucrose while dotted lines and pill cartoon indicated rearing on antibiotic. The line below shows the time line of the experiments and CFU sampling is indicated by the plates. Infection of CxSm1^RifR^ and CxSm2^SmR^ strains into antibiotic-treated or -untreated *Ae. aegypti* (**b**). Rifampamicin or spectinomycin was administered to mosquitoes in a sugar meal (capsule indicates antibiotic treatment). For both **a** and **b**, the upper panel shows a schematic of experimental design. The line graph indicates the titer of *Serratia* in mosquitoes, and the pie graph indicates infection prevalence in mosquitoes. For each time point, ten mosquitoes were sampled. Letters indicate significance from the Mann–Whitney test comparing density within a time point. Asterisks indicate a significant difference in *Serratia* in *Cx. quinquefasciatus* to *Ae. aegypti* (**a**) or antibiotic- and nonantibiotic-treated mosquitoes (**b**) using a Mann–Whitney test for densinty Fisher’s exact test for prevalence. *p < 0.05, **p < 0.01, ***p < 0.001, ****p < 0.0001. Both experiments (**a**, **b**) were repeated three times and comparible results were seen between replicates.

We investigated the ability of *Serratia* to colonize the novel *Ae. aegypti* host by reinfecting bacteria into mosquitoes in a 10% sucrose meal and monitored infection dynamics in the mosquito over time. The *Serratia* infection was completely lost from *Ae. aegypti* by 12 dpi, whereas the bacterial prevalence in the native host, *Cx. quinquefasciatus*, remained constantly high with infection levels ranging from 100% infection for CxSm1^RifR^ to 80% infection for CxSm2^SmR^ at 12 dpi (Fig. 1a). Of the mosquitoes that were infected, both *S. marcescens* strains infected *Ae. aegypti* (Galveston) at significantly lower densities compared to their native host, *Cx. quinquefasciatus* (Fig. 1a). For example, at 3 dpi, we saw ~1000 times less *Serratia* in *Ae. aegypti* compared to *Cx. quinquefasciatus* (Fig. 1a). We also examined other culturable microbiota by plating mosquito homogenates on nonselective LB plates, and in general, we saw few changes in the number of CFUs between groups in either *Ae. aegypti* or *Cx. quinquefasciatus* (Fig. S3), suggesting *Serratia* infection had minimal effect on the total bacterial load of culturable microbiota in mosquitoes. The inability of *Serratia* to persistently infect *Ae. aegypti*, which was not observed for other bacteria (Fig. S3), suggests that barriers, either of bacterial or host origin, were promoting the maladaption between these *Serratia* strains and this line of *Ae. aegypti*.

### Microbial interaction in the mosquito gut

To gain insights into the mechanism promoting the incompatibility between *Serratia* and *Ae. aegypti*, we repeated infections in antibiotic-treated mosquitoes as we speculated that the native microbiota of mosquitoes might interfere with the colonization of the host (Fig. 1b). We formulated this hypothesis as we have previously seen evidence of bacterial exclusion of symbiotic microbes in mosquitoes [4, 18]. Strikingly, both CxSm1^RifR^ and CxSm2^SmR^ colonized mosquitoes at significantly higher titers when mosquitoes were treated with antibiotics compared to mosquitoes reared conventionally without antibiotics (Mann–Whitney test; CxSm1^RifR^; day 3 *p* < 0.002, day 9 *p* < 0.01; day 12 *p* < 0.0001, CxSm2^SmR^; day 3 *p* < 0.03, day 9 *p* < 0.01; day 12 *p* < 0.01) (Fig. 1b). Furthermore, for both *Serratia* strains, significantly more mosquitoes were infected at day 12 in antibiotic-treated mosquitoes compared to untreated (Fisher’s exact test; CxSm1^RifR^ *p* = 0.01, CxSm2^SmR^ *p* = 0.0007). The levels of *Serratia* in *Ae. aegypti* after microbiome homeostasis that were disrupted by antibiotics were comparable to infections in the native host *Cx. quinquesfasciatus* (Fig. 1a). These data indicated that the *Ae. aegypti* (Galveston) line had the capacity to harbor *Serratia*, and that the incompatibility in mosquitoes with an intact microbiome (Fig. 1a, [4]) was due to members of the native microbiota inhibiting *Serratia*, as opposed to intrinsic host factors or genetic factors of the *S. marcescens* strains.

**Fig. 2:**
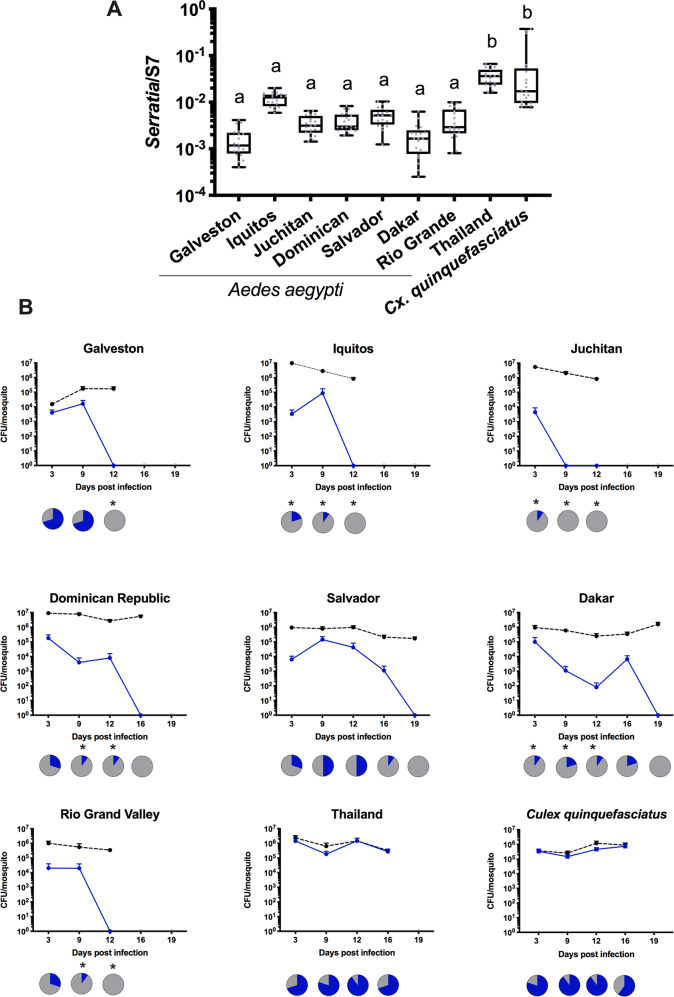
*Serratia* infection in diverse *Ae. aegypti* lines. The density of *Serratia* was determined by qPCR in eight *Ae. aegypti* lines (**a**). CxSm1^RifR^ was infected into Ae. aegypti lines and density (blue line) monitored over time (**b**). Total cultural microbiota (dotted line) was also quantified by culturing bacteria from homogenized mosquitoes on nonselective LB plates. Line graphs indicate titer of *Serratia* in mosquitoes, and pie graphs indicate infection prevalence in mosquitoes. Asterisks indicate a significant difference in *Serratia* prevalence in *Ae. aegypti* lines compared to *Cx. quinquefasciatus* using a Fisher’s exact test compared to the *Cx. quinquefasciatus* control line.

To determine how widespread these microbial interactions were in *Ae. aegypti* mosquitoes, we investigated eight diverse lines for native *Serratia* infections and their capacity to be infected with CxSm1^RifR^. When examining the native *Serratia* load by qPCR, seven of the eight *Ae. aegypti* lines had significantly lower titers compared to *C. quinquefasciatus* (Fig. 2a). Intriguingly, an *Ae. aegypti* line from Thailand had a high *Serratia* load that was comparable to the infection in the native *Culex* host. We also quantified *Serratia* levels in two other *Cx. quinquefasciatus* lines and found similar or higher loads of *Serratia* in these other lines (Fig. S4), indicating that the robust infection of *Serratia* in *Cx. quinquefasciatus* was commonplace. We then infected the CxSm1^RifR^ *Serratia* strain into these eight diverse *Ae. aegypti* lines. For these infections we focused our attention on CxSm1^RifR^, as overall, it appears this strain had a greater capacity to infect *Ae. aegypti* compared to CxSm2^SmR^. We therefore hypothesized that this strain would be more likely to infect non-native hosts. Similar to our previous experiments, *Serratia* poorly infected the Galveston line and was eliminated by 12 dpi. In the other lines, we saw some variation in the time it took for *Serratia* to be eliminated, with clearance occurring rapidly in the Juchitan and Iquitos lines. Whilst the process took longer in others (Dakar, Salvador and Dominican Republic), infection was ultimately cleared from all lines. In stark contrast to these seven lines, *Serratia* effectively colonized the Thailand line and the *Cx. quinquefasciatus* at high density and prevalence. Combined, the qPCR and re-infection experiments indicated the majority of *Ae. aegypti* lines that were not permissive to *Serratia* infection, but infection dynamics in the Thailand line were similar to the native *Culex* host.

### Microbiome analysis of resistant and susceptible mosquitoes

To determine which specific microbiota of *Ae. aegypti* altered *Serratia* infections, we sequenced the microbiome of four select lines that varied in the capacity to harbor the bacterium. The V3-V4 variable region of the 16S rRNA gene was sequenced from the Juchitan, Galveston, and Iquitos line, which were recalcitrant to *Serratia*, and the Thailand line that was able to sustain the infection similar to the native host. For each line, we sequenced 15 individuals and, on average, obtained 32,000 reads per mosquito. Rarefaction curves indicated that sufficient depth was obtained in the sequencing to adequately characterize the microbiome, while our spike in controls constituted 99.5% of the relative abundance indicating that there was negligible contamination in our sequencing (Fig. S5). Across all mosquito lines, we identify a total of 1163 bacterial OTUs, but only 55 were present in mosquitoes at an infection frequency above 1% (Table S5).

**Fig. 3:**
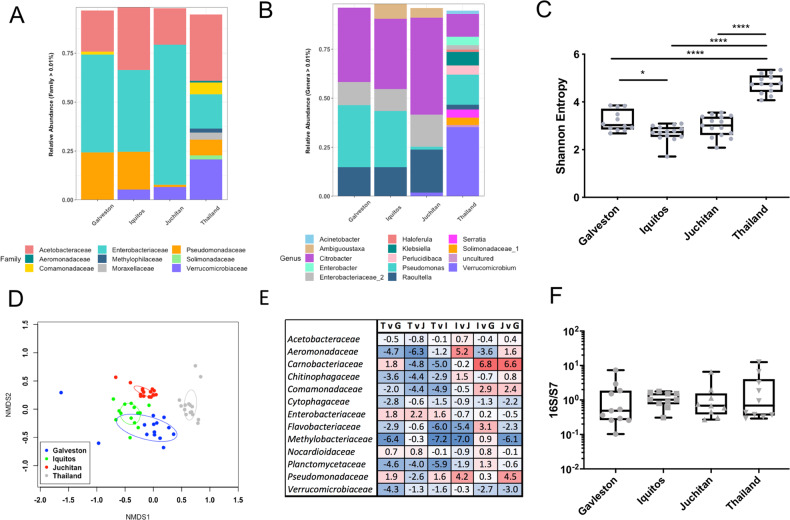
Microbiome analysis of the Galveston, Juchitan, Iquitos, and Thailand *Ae. aegypti* lines. 16S rRNA amplicon sequencing was done on female adult mosquitoes 5 days post eclosion. All mosquitoes were reared in the same laboratory environment under identical conditions. The relative abundance of bacterial communities at the family (**a**) and genus level **(b**). Alpha (Shannon’s entropy; *p < 0.05, ****p < 0.0001) (**c**) and beta (NMDS) (**d**) diversity metrics. Differential abundance analysis (ANCOM) of bacterial families in pairwise comparisons (**e**) between the four lines (T—Thailand, G—Galveson, J—Juchitan, I—Iquitos). A bolded value indicates a significant difference. Positive value indicates greater adundance of bacteria in the denominator, negative indicates greater number of bacteria in the numerator in the pairwise comparison. Total bacterial load in mosquito lines measured by qPCR (**f**).

When examining taxa within the microbiome, the majority of sequences were from the Proteobacteria, while others were associated with Verrucomicrobia and Bacteroidetes. Within the Proteobacteria, the most abundant OTUs were in with the families *Enterobacteriaceae, Acetobacteriaceae, and Pseudomonasaceae*, while the Thailand line harbored a considerable amount of *Verrucomicrobiaceae* compared to the other three lines (Fig. 3a). Confirming our qPCR data, we saw minimal or no *Serratia* infection in the Galveston, Iquitos, or Juchitan lines, but this bacterium comprised ~4% of the relative abundance of the Thailand line (Fig. 3b). It was also noticeable that the Thailand line possessed a higher diversity of OTUs compared to the other lines (Fig. S6 and Table S5). This was corroborated by alpha diversity measures, which indicated that the Thailand line had a significantly elevated Shannon’s diversity index compared to the other three lines (Fig. 3c). To examine the community structure of the microbiome in each line, we undertook NMDS analysis based on Bray–Curtis dissimilarity. Strikingly, the microbiomes of each line were significantly different from each other (Fig. 3d, *p* < 0.05); however, it was evident from the clustering that the Thailand line was considerably divergent compared to the other three lines.

To examine specific taxa that may be the cause of microbial incompatibility, we undertook pairwise comparisons to identify bacteria that were differentially abundant between lines. We examined differences at the family level using ANCOM, which is specifically designed to handle variable microbiome data [57]. While the abundance of several families was significantly different between lines, the *Enterobacteriaceae* was the only family that was consistently reduced in the Thailand line compared to the other three lines (Fig. 3e). In addition to amplicon sequencing, we used qPCR to determine the total microbial load of mosquitoes and found that each possessed a similar density of bacteria (Fig. 3f), indicating the increase in taxa in the Thailand line were not simply due to possessing a greater number of bacteria. Taken together, these data indicated that the microbiome of the Thailand line was substantially different from the other lines and that members of the *Enterobacteriaceae* can inhibit *Serratia* infection in mosquitoes.

### Co-infections in gnotobiotic infection model

**Fig. 4:**
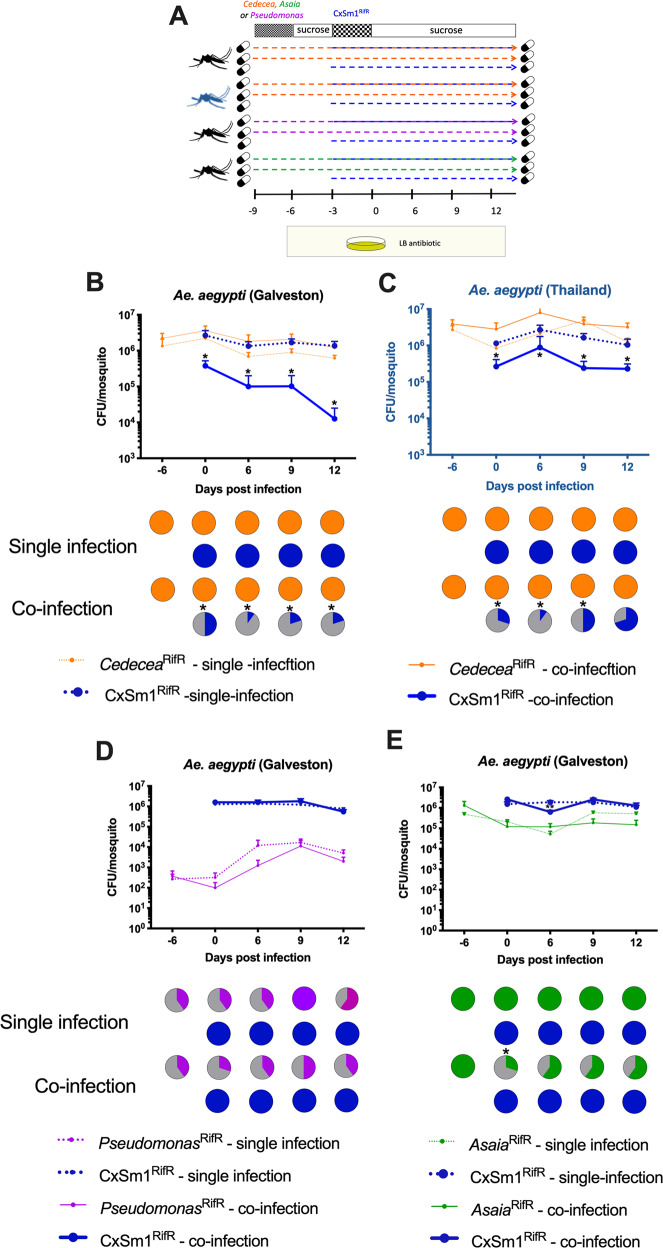
Co-infection of *Enterobacteriaceae* and *Serratia* in *Ae. aegpyti*. Schematic depicting the co-infection experimental design **a** Dotted lines and pill cartoon indicated rearing on antibiotic. The mosquito color indicates genotype (black: Galveston line, blue: Thailand line). Bacterial administration protocols and sampling time are represented above and below, respectively. Co-infection of *Cedecea*^RifR^ and CxSm1^RifR^ in *Ae. aegpyti* (Galveston) (**b**) and Thailand (**c**) lines. Control co-infections whereby *Pseudomonas* (**d**) or *Asaia* (**e**) were infected prior to CxSm1^RifR^. Line graphs show bacteria density (CFU/mosquito), and pie graphs show infection prevalence. For each time point, ten mosquitoes were sampled. Letters indicate significance from ANOVA comparing density within a time point. Asterisks indicate a significant difference between *Serratia* prevalence in single and co-infected mosquitoes using a Fisher’s exact test.

To functionally demonstrate that members of the *Enterobacteriaceae* interfere with *Serratia* colonization, we undertook a series of co-infection experiments in antibiotic-treated mosquito lines. Prior to infection of the CxSm1^RifR^ *Serratia* strain, we infected mosquitoes with *Cedecea*^RifR^, a member of the *Enterobacteriaceae* that commonly infects mosquitoes, or other *Acetobacteraceae* and *Pseuduomonadaceae* bacteria as controls (Fig. 4a). We chose *Cedecea* as we have previously documented that this bacterium infects *Ae. aegypti* effectively [4]. The infection prevalence of *Serratia* in the co-infected *Ae. aegypti* Galveston line was significantly reduced in all time points (Fig. 4b, *p* < 0.05, the Fisher’s exact test). In the few mosquitoes that did harbor a *Serratia* infection, the density was significantly lower compared to the single infection (Fig. 4b, *t*-test *p* < 0.05). These data indicated *Serratia* colonization was inhibited by the presence of *Cedecea*, and the phenotype we observed previously in conventionally reared mosquitoes could be recapitulated in a gnotobiotic setting. Similarly, we also found that the prevalence of *Serratia* was reduced by co-infection in the *Ae. aegypti* Thailand line (*p* = 0.05, Fisher’s exact test), although this effect was more subtle, and no significant difference was observed at 12 dpi (Fig. 4c). In contrast to co-infection with *Cedecea*, we found no effect in *Serratia* prevalence or titers when co-infected with *Asaia* or *Pseudomonas* (Fig. 4e, f), which are members of the *Acetobacteraceae* and *Pseduomonadaceae* families, respectively. Interestingly, there was evidence that *Serratia* interferes with *Asaia* infections in *Ae. aegypti, as* there was an initial reduction in the prevalence of *Asaia* in the co-infected group compared to the single infection (Fig. 4f, *p* = 0.05, Fisher’s exact test). Together, these co-infection studies demonstrate that inhibition of *Serratia* colonization in *Ae. aegypti* is bacteria-specific, and that antagonism occurs between members of the *Enterobacteriaceae* and *Serratia*.

**Fig. 5:**
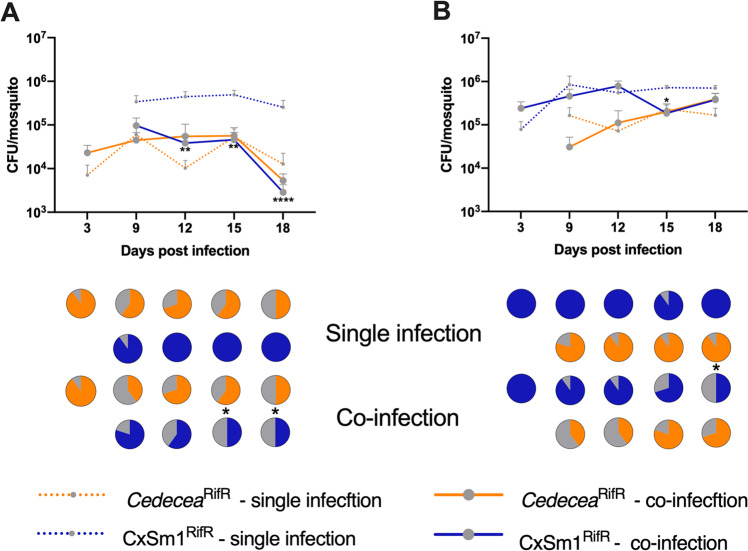
Co-infection of *Cedecea* and *Serratia* in *Cx. quinquefasciatus*. Infection of *Cedecea* followed by CxSm1^RifR^ (**a**) or CxSm1^RifR^ followed by *Cedecea* (**b**) in *Cx. quinquefasciatus*. Line graphs show bacteria density (CFU/mosquito), and pie graphs show infection prevalence. For each time point, ten mosquitoes were sampled. Letters indicate significance from Mann–Whitney test comparing density of CxSm1^RifR^ single and co-infectons within a time point. For prevalence data, asterisks indicate a significant difference between *Serratia* prevalence in single and co-infected mosquitoes using a Fisher’s exact test. *p < 0.05, **p < 0.01, ****p < 0.0001.

To determine how *Cedecea* influenced *Serratia* in its native host, we repeated co-infection experiments in *Cx. quinquefasciatus* using the gnotobiotic infection model. *Cedecea* infected *Culex* mosquitos less effectively compared to *Aedes*, with infection densities around two logs lower and an infection prevalence that dropped to 50% over the course of the experiment (Fig. 5). Despite a lower level of infection, *Cedecea* infection prior to *Serratia* reduced the infection of the latter. At 15 and 18 dpi, the prevalence of *Serratia* in the co-infection was 50% compared to 100% in the single infection (Fig. 5a, *p* = 0.03, Fisher’s exact test). We also examined the effect of *Cedecea* on an established *Serratia* infection by reversing the order each bacterium was administered to the mosquito. In this case, the prevalence of *Serratia* in the co-infection was significantly reduced only at the 18 dpi time point (Fig. 5b, *p* = 0.03 Fishers exact test). Taken together, these data show that antagonism between *Serratia* and other *Enterobacteriaceae* also occurs in *Culex* mosquitoes.

### Effect of *Serratia* exposure on blood-feeding behavior

**Fig. 6:**
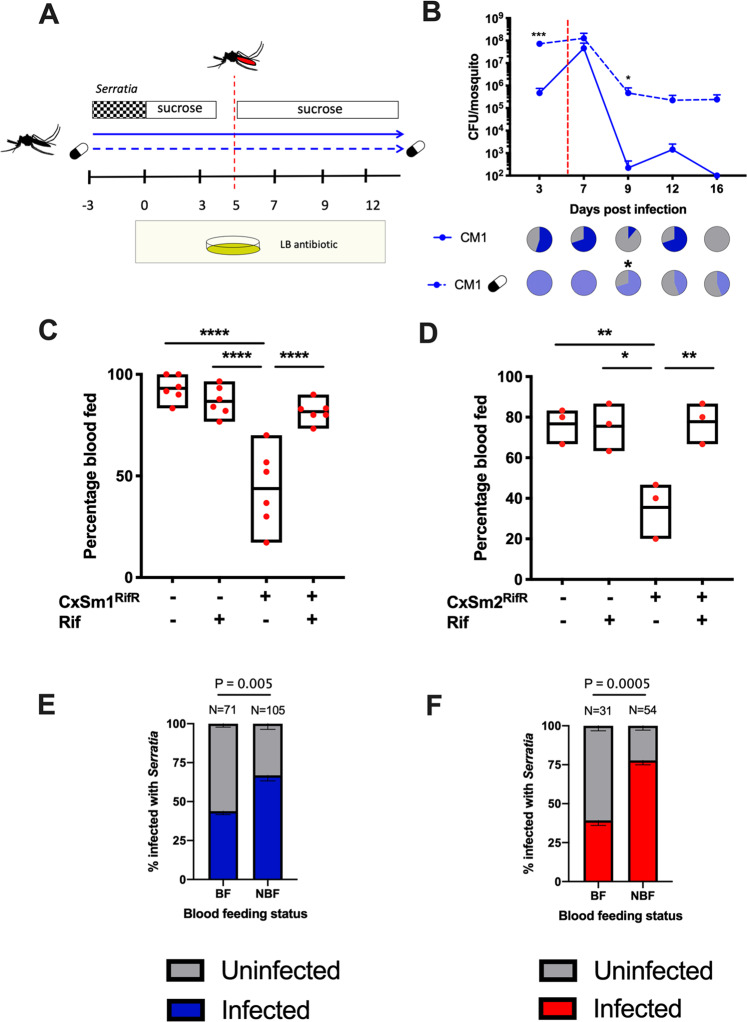
Interaction between *Serratia* infection and blood feeding in *Ae. aegypti*. Schematic depicting the infection and blood-feeding experimental design (**a**). Infection density and prevalence of CxSm1^RifR^ in conventional and antibiotic-fed *Ae. aegypti* (**b**). The red dotted line indicates the timing of blood meal. Significance was determined using a *T*-test comparing conventional and antibiotic groups for each time point. Ten mosquitoes were examined at each time point. The capsule indicates antibiotic treatment. Percentage of mosquitoes to take a blood meal for CxSm1^RifR^ (**c**) or CxSm2^SmR^ (**d**) infected or uninfected mosquitoes. Significance was determined using a one-way ANOVA with Tukey’s multiple comparisons test, *p < 0.05, **p < 0.01, ****p < 0.0001. Either six (**c**) or three (**d**) cups were used for feeding experiments with 50 mosquitoes per cup. The CxSm1^RifR^ experiment was replicated twice (three cups each and data pooled). Percentage *Serratia* administered mosquitoes infected with CxSm1^RifR^ (**e**) or CxSm2^SmR^ (**f**) blood-fed, or non-blood-fed groups. Fisher’s exact test was used to determine significance. Samples size is indicated for each group above the bars.

Anautogenous mosquitoes require a blood meal to acquire nutrition for egg development. Ingested blood alters the gut microbiota composition and abundance, often increasing total bacterial load but decreasing species richness [67, 68]. In other mosquito species, *Serratia* has been seen to rapidly increase in titer after a blood meal [69–71] and, in some cases, can be lethal to the host [38]. As such, we investigated the influence of blood feeding on *Serratia*-infected *Ae. aegypti* (Fig. 6a). We measured bacterial load in the mosquito (Fig. 6b) as well as a range of life history traits. For these experiments, we focused our attention on CxSm1^RifR^. In contrast to a previous study [38], we observed no fitness costs to infection in terms of mosquito survival pre- or post-blood meal (Fig. S7). After a blood meal, *Serratia* density precipitously increased around 100-fold. The increase in the antibiotic-treated mosquitoes was more subtle, likely because the bacterial load was initially greater, suggesting that there is an upper limit to infections. After blood feeding, *Serratia* infections were comparable to densities and infection frequencies seen in sugar-fed mosquitoes (Figs. 1 and 4), with levels in antibiotic-treated mosquitoes being maintained at around 1 × 10^6^ bacteria/mosquito. In conventionally reared mosquitoes, *Serratia* was eliminated, albeit over a longer time period, likely due to the increased density of the bacterium after stimulation from the blood meal. Post blood feeding, *Serratia* densities equilibrated to levels around 10^6^, which were comparable to infection densities seen in non-blood-fed mosquitoes (Fig. 6b). While we saw no differences in egg number (Fig. S8), in the process of conducting these experiments, we observed that CxSm1^RifR-^infected mosquitoes were less inclined to take a blood meal when reared on a convention sugar diet.

We therefore investigated further whether *Serratia* infection altered mosquito blood-feeding behavior. After providing mosquitoes with the opportunity to feed, we saw that significantly fewer females had imbibed a blood meal compared to uninfected or antibiotic-treated CxSm1^RifR^ infected mosquitoes (Fig. 6c, ANOVA *p* < 0.001). Blood-feeding rates in *Serratia*-infected *Ae. aegypti* were restored when mosquitoes were fed antibiotics, indicating these behavioral changes were mediated by the interplay between CxSm1^RifR^ and other bacterial constituents of the microbiome susceptible to antibiotics. Given this intriguing finding, we repeated these experiments with the CxSm2^SmR^ isolate. Similar to findings with its close relative, the CxSm2^SmR^ *Serratia* strain altered the blood-feeding rates in mosquitoes (Fig. 6d, ANOVA *p* < 0.001). Given the heterogeneity in the prevalence of CxSm1^RifR^ and CxSm2^SmR^ in conventionally reared mosquitoes, we examined individuals that did or did not blood feed for *Serratia* infection. For both CxSm1^RifR^ (Fig. 6e) and CxSm2^SmR^ (Fig. 6f), the *Serratia* infection rate was significantly higher in non-blood-fed mosquitoes compared to blood-fed (CxSm1^RifR^ *p* < 0.005; CxSm2^SmR^ *p* < 0.005), indicating that mosquitoes that took a blood meal were less likely to be infected with *Serratia*. When considering this, it is likely that the reductions we observed at the population level (Fig. 6c, d) are conservative, and the effect of *Serratia* infection on blood-feeding behavior is more pronounced.

## Discussion

The interplay between the host and microbes can dictate insect microbiome homeostasis, but little is known regarding how microbe–microbe interactions within the gut influence microbial composition and abundance. Previously we identified a *Serratia* infection gradient in the arboviral vectors, *Ae. aegypti*, *Ae. albopcitus*, and *Cx. quinquefasciatus*, with high loads in the latter and an absence of infection in the former [4]. Here we show that *Serratia* poorly infects many *Ae. aegypti* strains and that the mechanism mediating this incompatibility is competitive exclusion with *Cedecea*, a member of the *Enterobacteriaceae* and close relatives of *Serratia* (also a member of the Gammaproteobacteria and the *Enterobacterales*, but the family *Yersiniaceae*). Given that *Serratia* can influence vector competence in mosquitoes and has been proposed as a microbe for paratransgenic control [69, 70], it is imperative we enhance our understanding of factors that influence *Serratia* acquisition in the mosquito gut.

After confirming that members of the microbiota were inhibiting *Serratia* colonization of mosquitoes, we characterized the microbiome of *Ae. aegypti* lines susceptible and resistant to infection. Interestingly, the susceptible Thailand line possessed a distinct and species-rich microbiome, and had significantly lower levels of *Enterobacteriaceae*. We speculated that this line had lost its capacity to maintain microbiome homeostasis, which subsequently enabled numerous other bacterial species to colonize. These other species likely reduced the abundance of *Enterobacteriaceae* in the host, as our qPCR data indicated that the Thailand line had a similar total bacterial load compared to the other lines. The reduced levels of potentially antagonistic *Enterobacteriaceae* in the Thailand line enabled the colonization of the *Serratia* at levels similar to *Culex* mosquitoes. This theory is strongly supported by the fact that inhibition of *Serratia* was restored in the Thailand line when mosquitoes were pre-infected with *Cedecea*.

Microbiome dysbiosis can profoundly alter several host phenotypes in insects, including symbiont processes [18]. In the Oriental fruit fly, *Bactrocera dorsalis*, suppression of the dual oxidase gene (BdDuox) led to microbiome dysbiosis and an overabundance of *Verrucomicrobiaceae* bacteria [72]. Increases in V*errucomicrobiaceae* have also been observed in mammalian systems when the microbiome transfers to a dysbiotic state [73–76]. In our analysis*, Verrucomicrobiaceae* was a dominant member of the microbiome of the Thailand line, yet was at relatively low abundance in the Iquitos and Juchitan lines and barely detectable in the Galveston line. The presence of this family suggests that the microbiome of the Thailand line was in a state of dysbiosis. In the Galveston line, the co-infection experiments recapitulated our previous results indicating that bacterial co-exclusion was the main factor driving *Serratia* incompatibility. However, the effects in the Thailand line were more subtle, with the presence of *Cedecea* only reducing the *Serratia* infection prevalence at earlier time points and not influencing titer. This suggests that other host factors likely contribute to the incompatibility of *Serratia* in the Galveston line, but these factors were deficient in the Thailand line, resulting in the more subtle phenotype.

While there are examples that a broad range of microbes can influence feeding behavior in insects, relatively little is known regarding how gut-associated microbes contribute to these phenotypes. Our data indicate that *Serratia* acts in concert with other microbes to reduce blood feeding. There is a complex immune interplay between gut microbes and the host [77–79], and it is possible that disruption of microbiome homeostatis by *Serratia* infection may alter basal immunity which subsequently affects feeding behavior. Alternatively, these mosquitoes may be suffering the effects of infection or microbiome dysbiosis resulting in a lack of interest in feeding. However, similar to findings in *Culex* mosquitoes [80], we saw little evidence for *Serratia* affecting other life history traits. Further studies are required to decipher the exact mechanism(s) causing this phenotype. From a vector control standpoint, reducing blood-feeding rates will greatly influence pathogen transmission. However, this phenotype is mediated by an interaction between *Serratia* and other native microbes of the *Ae. aegpyti*. Given the inherent variability in the microbiome of mosquitoes, further investigations are warranted to determine how universal this phenotype is, and in general how alterations in mosquito behaviors caused by microbiome dysbiosis can then impact upon vectorial capacity. In the laboratory setting, reduced feeding rates would act as a distinct process to eliminate *Serratia* infections from the microbiome of *Ae. aegypti*.

Another important aspect of our work is the finding that *Ae. aegpyti* lines reared under uniform insectary conditions have diverse microbiomes. While it was evident that the Thailand line has a particularly divergent microbiome, the microbiomes of the Juchitan, Galveston, and Iquitos lines were also distinct from each other. This is contrary to a recent finding [41], and suggests that similarity in microbiomes driven by environmental factors is not universal, and host or bacterial factors also play a role in microbiota community assembly and can lead to microbiome divergence, as shown in *Drosophila* [81, 82]. Here we demonstrate in mosquitoes that host genotype profoundly alters bacterial microbiome composition.

In conclusion, we show that microbe–microbe interactions influence microbiome composition and abundance in mosquito vectors. These processes are robust and can prevent the transfer of microbiota between mosquitoes that share a common environment by distinct mechanisms. Transfer of microbiota can occur in a host when microbiome homeostasis is disrupted, but this can also alter phenotypes important for host biology. Furthermore, we show that microbiota transfer can change mosquito traits that are important for pathogen transmission. From an applied standpoint, a greater understanding of the factors dictating microbial exclusion and acquisition could be exploited to develop strategies to create mosquitoes with designer microbiomes that induce desirable properties for vector control.

## Supplementary information

Supplementary material

Table S5
