# Supplementary material for: Microbial interactions in the mosquito gut determine Serratia colonization and blood-feeding propensity
Source: ISME J. 2020 Sep 7;15(1):93–108. doi: 10.1038/s41396-020-00763-3 (PMC7852612; doi:10.1038/s41396-020-00763-3)
Supplement: Supplementary file 1 — Supplementary material [file 41396_2020_763_MOESM1_ESM.docx]

**SUPPLEMENTARY INFORMATION**

**Microbial interactions in the mosquito gut determine *Serratia* colonization and blood feeding propensity.**

^1^Department of Pathology, University of Texas Medical Branch, Galveston, TX, USA.

^2^Departments of Vector Biology and Tropical Disease Biology, Centre for Neglected Tropical Disease, Liverpool School of Tropical Medicine, Liverpool, UK.

^3^World Reference Center for Emerging Viruses and Arboviruses, Institute for Human Infections and Immunity, and Department of Microbiology and Immunology, University of Texas Medical Branch, Galveston, TX, USA,

^4^Department of Pharmacology and Toxicology, Sealy Center for Structural Biology, University of Texas Medical Branch, Galveston, TX, United States.

^5^Department of Paediatrics and Tropical Medicine, Baylor College of Medicine, Houston, TX, United States.

^6^The Institute for Translational Science, University of Texas Medical Branch, Galveston, TX, United States. Institute for Global Health and Translational Science and SUNY Center for Environmental Health and Medicine, SUNY Upstate Medical University, Syracuse, NY, United States.

^7^Institute of Integrative Biology, University of Liverpool, Liverpool, UK.

^8^Department of Biochemistry and Molecular Biology, University of Texas Medical Branch, Galveston, TX, USA,

^9^Departments of Vector Biology and Clinical Sciences, Liverpool School of Tropical Medicine, Liverpool, UK.

**^*^** These authors contributed equally.

^#^Corresponding author. Grant Hughes: [grant.hughes@lstmed.ac.uk](mailto:grant.hughes@lstmed.ac.uk)

**Supplementary figures.**


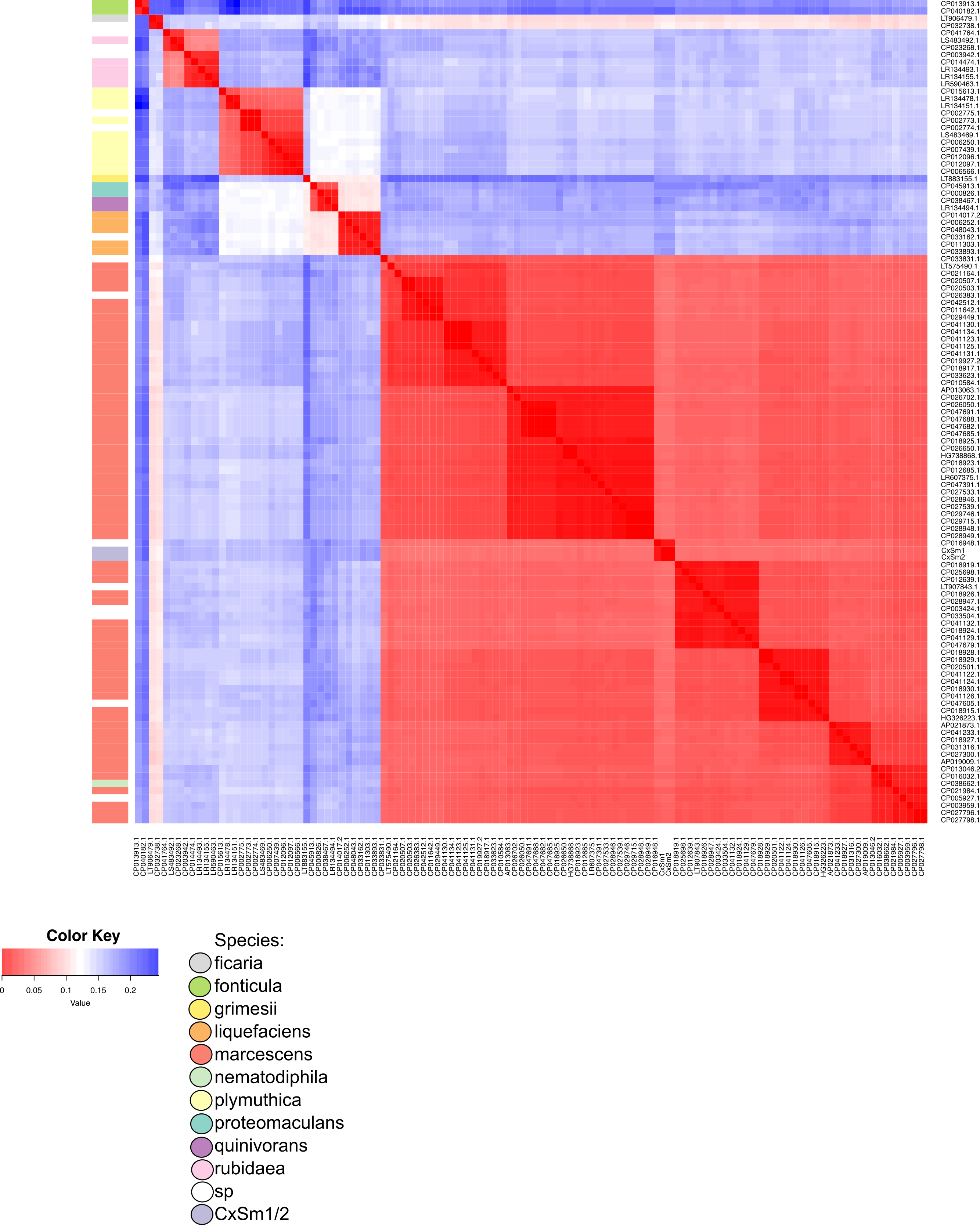


**Figure S1. Genomic analysis of CxSm1 and CxSm2 strains.** Our isolates were compared to a set of publicly available *Serratia* reference genomes (Table S3) using average nucleotide identity (ANI). The heatmap indicates pairwise comparisons of ANI in an all-against-all comparison, showing our isolates as a distinct subgroup within *S. marcescens*. Colour legends for the similarity (ANI) and the species designation as given in GenBank are detailed below (Table S3).


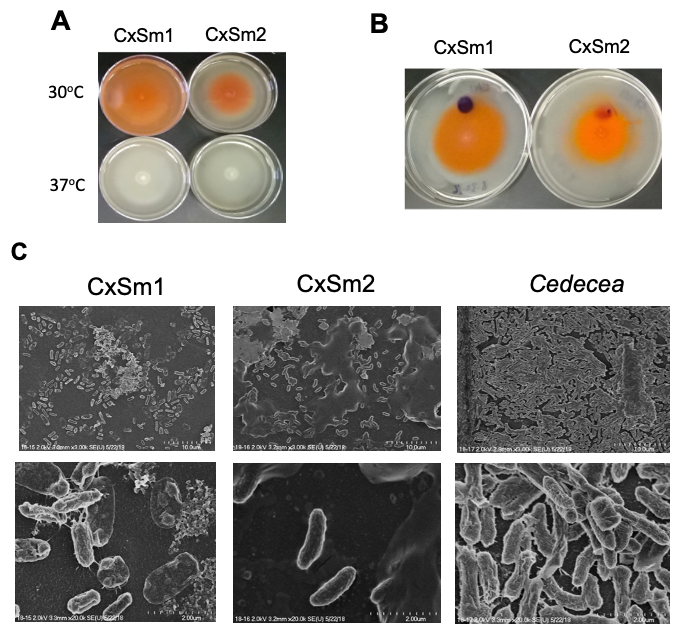


**Figures S2. Phenotypic characterisation of *Serratia* isolates.** Swimming motility of CxSm1 and CxSm2 at different temperatures (**A**). Oxidase activity at 30°C (**B**). Scanning electron microscopy of CxSm1, CxSm2, and *Cedecea*.

**
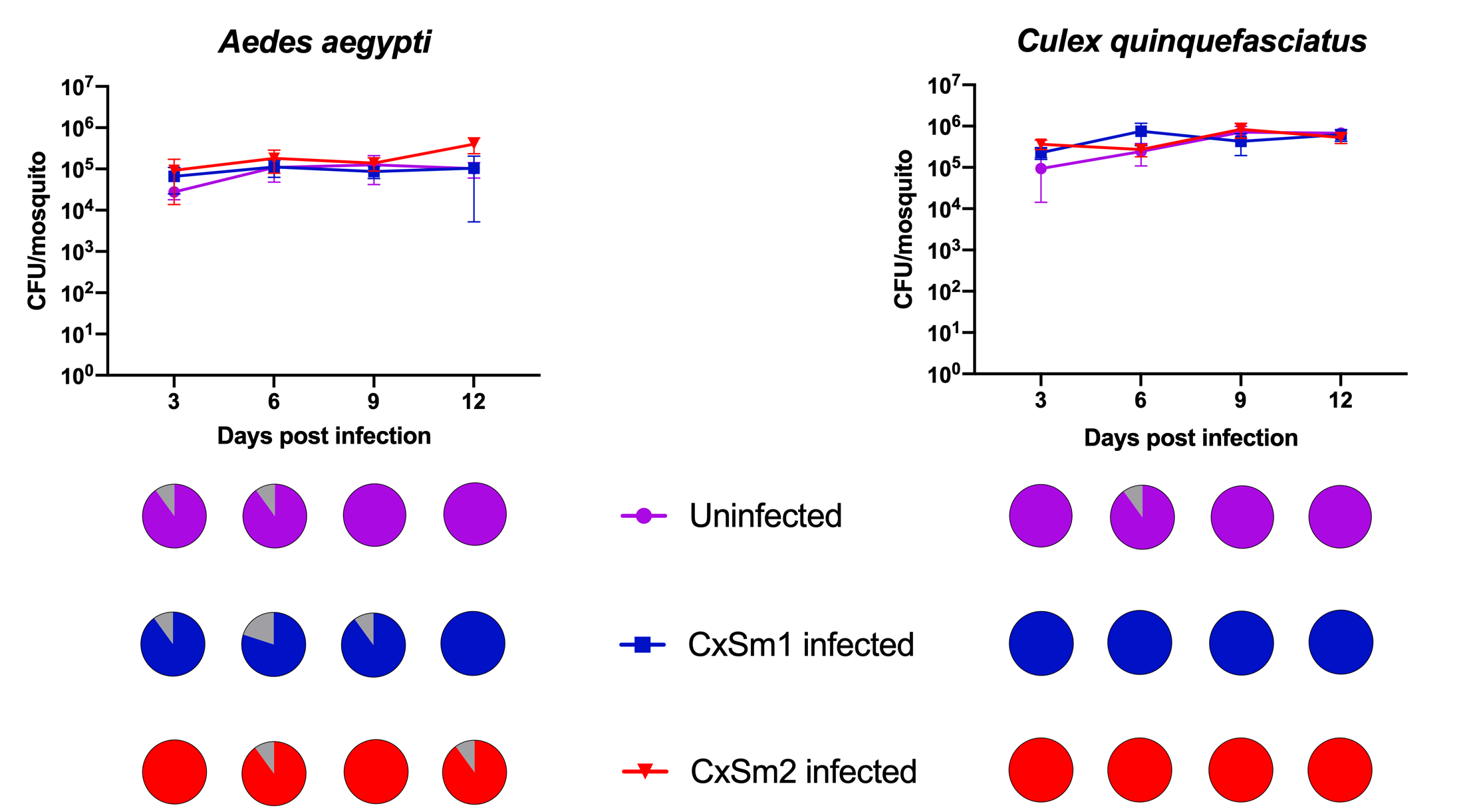
**

**Figure S3. Total culturable bacterial loads in mosquitoes.** Homogenized mosquitoes were plated on LB agar plates without selection. Line graph indicates bacterial density (CFU/mosquito) and pie graph indicated prevalence. Data related to Figure 1A.


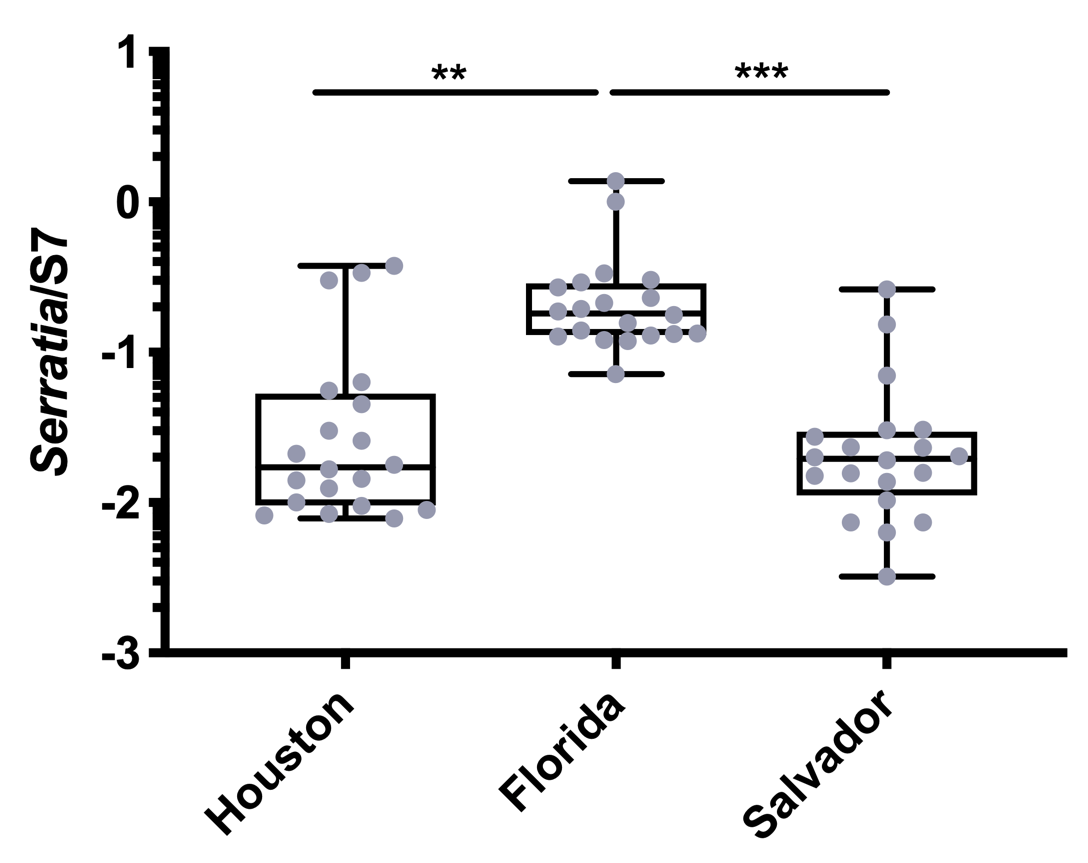


**Figure S4. Native *Serratia* densities in *Cx. quinquefasciatus* strains.** qPCR relative abundance (*Serratia*/S7) values of the Houston, Florida and Salvador *Cx. quinquefasciatus* lines reared under identical conditions in the same insectary. Data were analysed with an ANOVA with Tukeys multiple comparison. (** p < 0.01, *** p < 0.001).

**
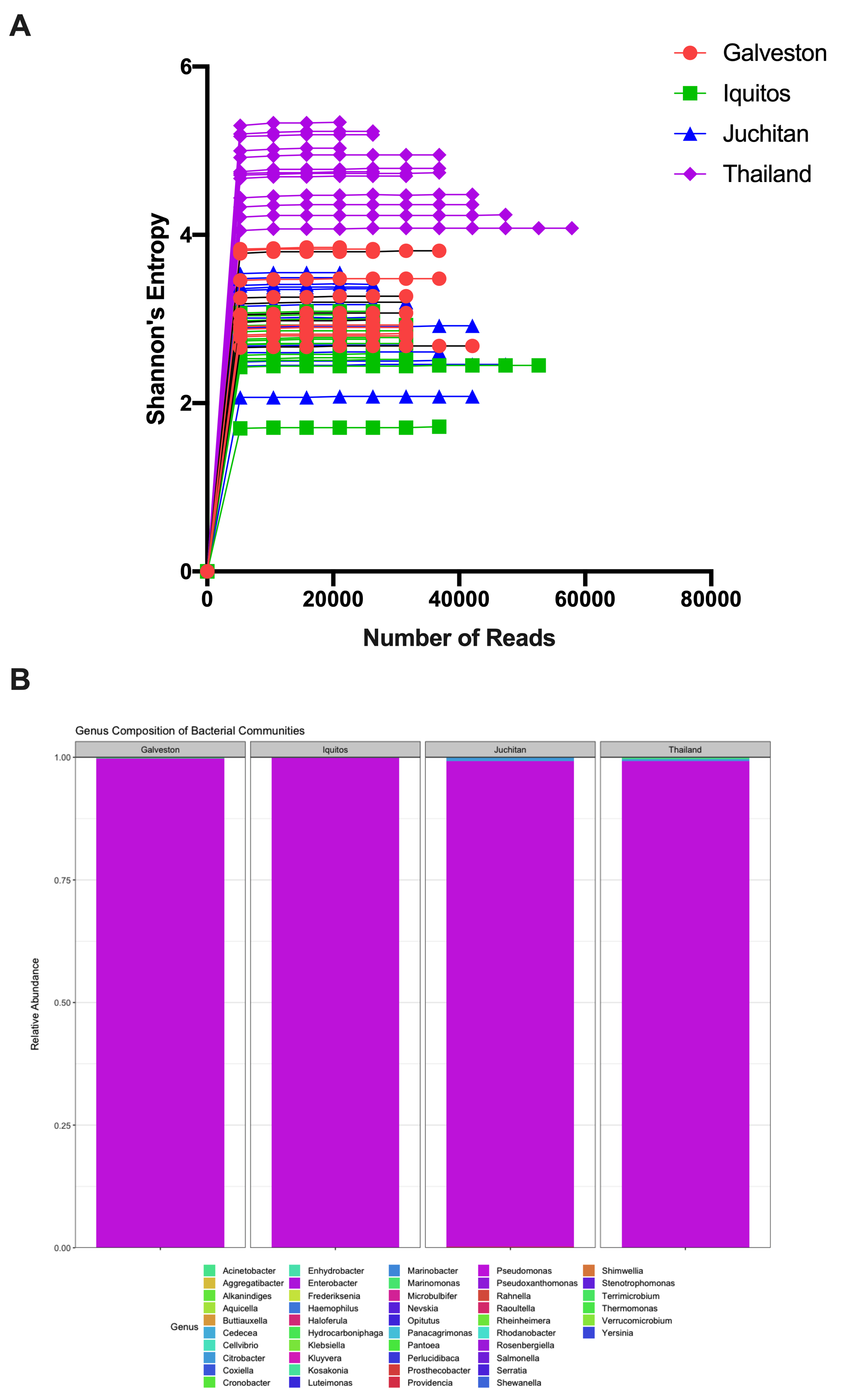
**

**Figure S5. Validation metrics of microbiome sequencing data.** Shannon entropy rarefied at intervals between 0 and 80000 reads for each individual (A). Relative abundance of positive spike-in controls (B).


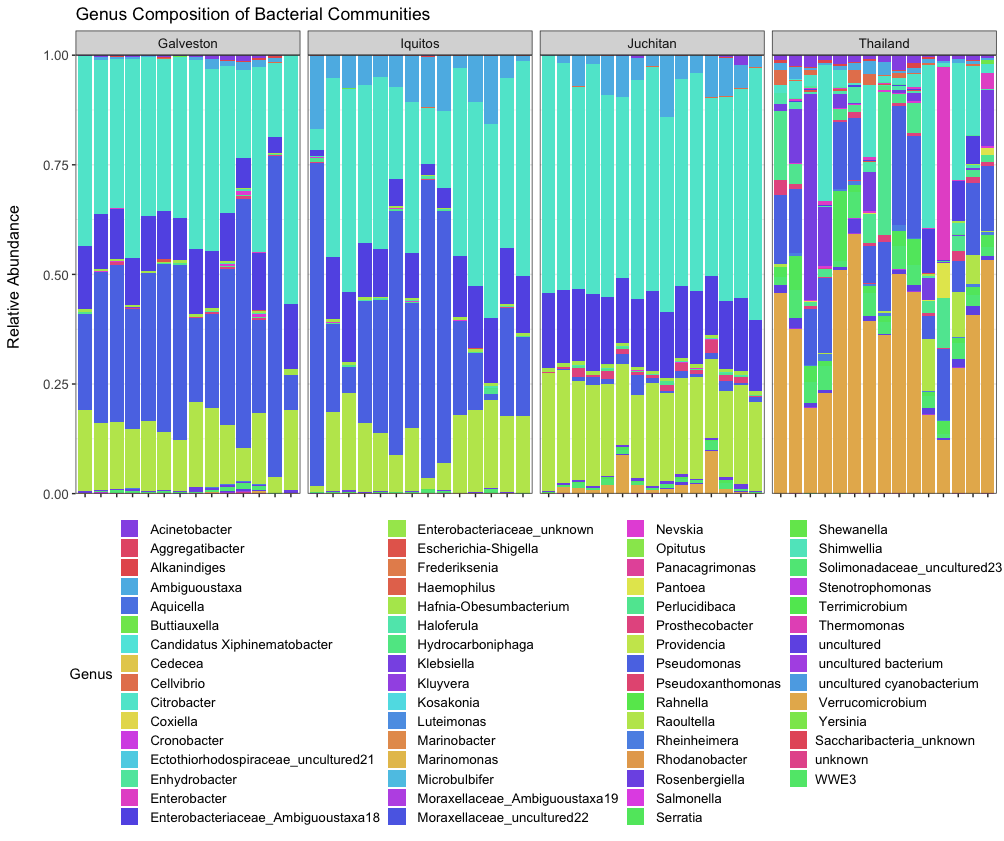


**Figure S6. Microbiome relative abundance measures for each individual at the genus levels.**

**
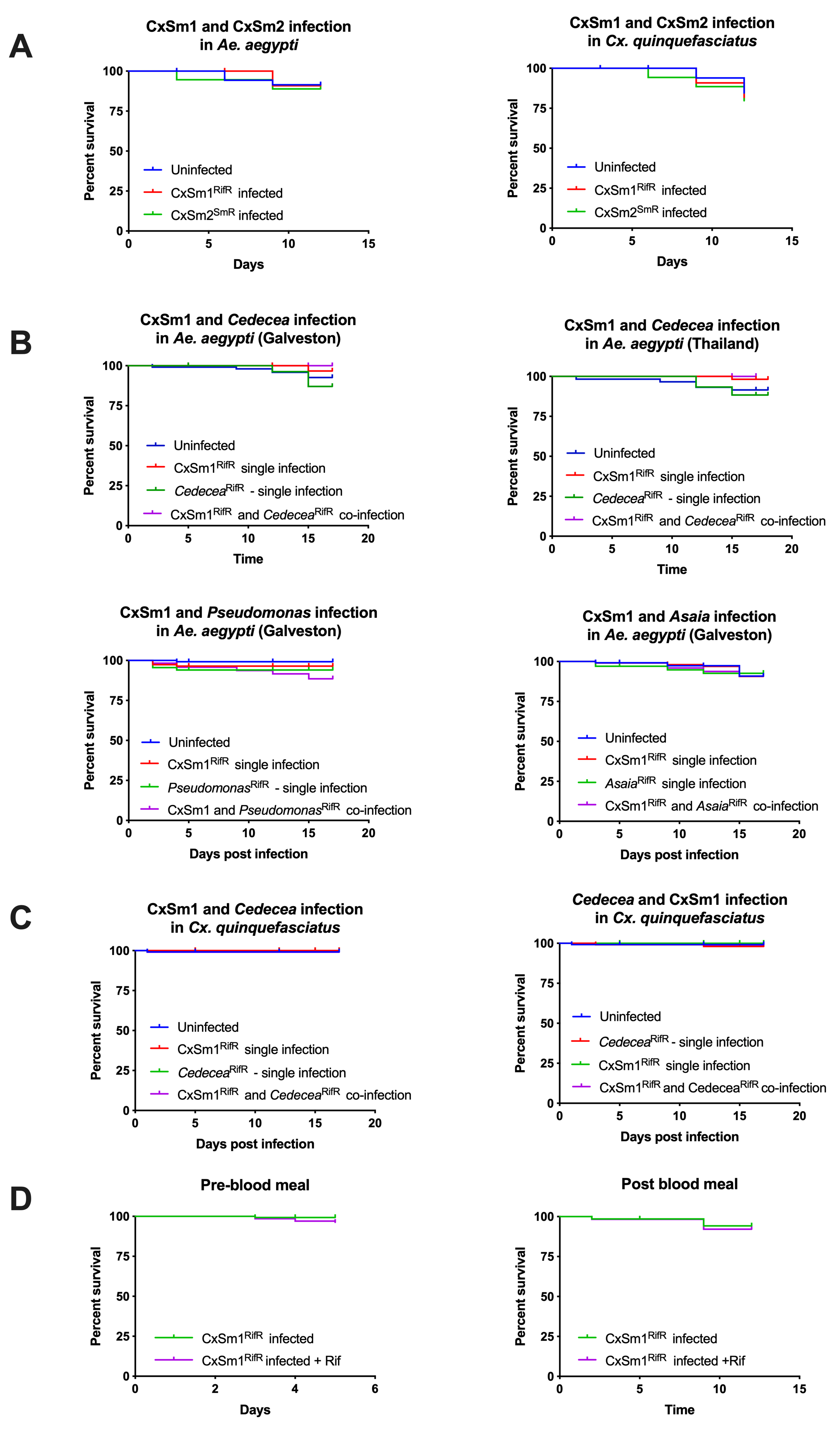
**

**Figure S7. Survival curves of *Serratia* infected mosquitoes. Curves** relate to Figure 1A (**A**), Figure 4 (**B**), Figure 5 (**C**), and Figure 6 (**D**).

**
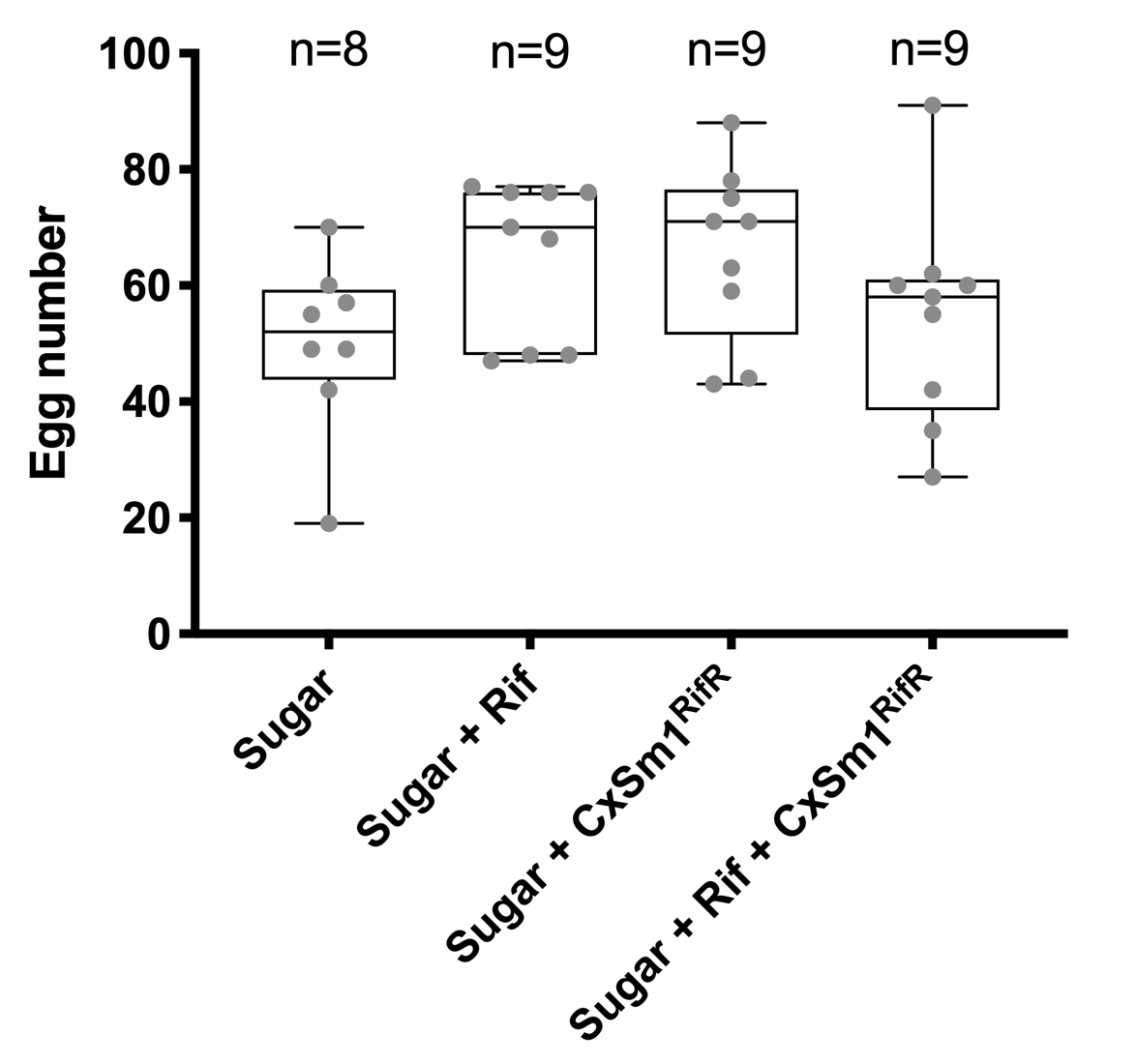
**

**Figure S8. Reproductive output of blood fed mosquitoes measured in terms of egg number.**

**Supplementary tables**

**Table S1. List of mosquito lines used in experiments.**

| List of generations number (or time) of Aedes aegpyti colonies | | | | |
| --- | --- | --- | --- | --- |
|  |  |  |  |  |
| Species | Colony | Age |  |  |
| Ae. aegypti | Galveston* | F4 |  |  |
| Ae. aegypti | Iquitos* | 10 years |  |  |
| Ae. aegypti | Rio Grand Valley | F7 |  |  |
| Ae. aegypti | Juchitan oaxaca* | 3 years |  |  |
| Ae. aegypti | Dakar | 4 years |  |  |
| Ae. aegypti | Dominican Republic | F10 |  |  |
| Ae. aegypti | Thailand* | 10 years |  |  |
| Ae. aegypti | Salvador | F8 |  |  |
|  |  |  |  |  |
| * indicates lines used from microbiome sequencing. | | | |  |
|  |  |  |  |  |

**Table S2. Primers used for PCR and qPCR.**

| **Gene** | **Forward primer (5’-3’)** | **Reverse primer (5’-3’)** | **Reference** |
| --- | --- | --- | --- |
| 16S rRNA | AGAGTTTGATCATGGCTCAG | GTGTGACGGGCGGTGTGTAC | 1 |
| psf1 | CCGGCATCGGCAAAGTCT | ATCTGGCCCGGCTCGTAGCC | 2 |
| aeg-S7 | ACCGCCG TCTACGATGCCA | ATGGTGGTCTGCTGGTTCTT | 3 |
| Cq-S7 | CTGGAGATGAACTGGACCT | CTT GTACACCGACGTGAAGG | 4 |

**Table S3. Selection of reference genomes used in comparative genomics.**

| Organism | Species | Strain | Accession |
| --- | --- | --- | --- |
| Serratia marcescens | marcescens | SM39 | AP013063.1 |
| Serratia marcescens | marcescens | AS-1 | AP019009.1 |
| Serratia marcescens | marcescens | ATCC 274 | AP021873.1 |
| Serratia proteamaculans | proteamaculans | 568 | CP000826.1 |
| Serratia plymuthica | plymuthica | AS9 | CP002773.1 |
| Serratia sp. | sp | AS12 | CP002774.1 |
| Serratia sp. | sp | AS13 | CP002775.1 |
| Serratia sp. | sp | SCBI | CP003424.1 |
| Serratia sp. | sp | FGI94 | CP003942.1 |
| Serratia marcescens | marcescens | WW4 | CP003959.1 |
| Serratia sp. | sp | FS14 | CP005927.1 |
| Serratia plymuthica | plymuthica | 4Rx13 | CP006250.1 |
| Serratia liquefaciens | liquefaciens | ATCC 27592 | CP006252.1 |
| Serratia plymuthica | plymuthica | S13 | CP006566.1 |
| Serratia plymuthica | plymuthica | V4 | CP007439.1 |
| Serratia marcescens | marcescens | AS1 | CP010584.1 |
| Serratia liquefaciens | liquefaciens | HUMV-21 | CP011303.1 |
| Serratia marcescens | marcescens | CAV1492 | CP011642.1 |
| Serratia plymuthica | plymuthica | 3Rp8 | CP012096.1 |
| Serratia plymuthica | plymuthica | 3Re4-18 | CP012097.1 |
| Serratia marcescens | marcescens | RSC-14 | CP012639.1 |
| Serratia marcescens | marcescens | SmUNAM836 | CP012685.1 |
| Serratia marcescens | marcescens | B3R3 | CP013046.2 |
| Serratia fonticola | fonticola | GS2 | CP013913.1 |
| Serratia liquefaciens | liquefaciens | FDAARGOS_125 | CP014017.2 |
| Serratia rubidaea | rubidaea | 1122 | CP014474.1 |
| Serratia plymuthica | plymuthica | PRI-2C | CP015613.1 |
| Serratia marcescens | marcescens | U36365 | CP016032.1 |
| Serratia sp. | sp | YD25 | CP016948.1 |
| Serratia marcescens | marcescens | UMH1 | CP018915.1 |
| Serratia marcescens | marcescens | UMH5 | CP018917.1 |
| Serratia marcescens | marcescens | UMH7 | CP018919.1 |
| Serratia marcescens | marcescens | UMH9 | CP018923.1 |
| Serratia marcescens | marcescens | UMH2 | CP018924.1 |
| Serratia marcescens | marcescens | UMH3 | CP018925.1 |
| Serratia marcescens | marcescens | UMH6 | CP018926.1 |
| Serratia marcescens | marcescens | UMH8 | CP018927.1 |
| Serratia marcescens | marcescens | UMH10 | CP018928.1 |
| Serratia marcescens | marcescens | UMH11 | CP018929.1 |
| Serratia marcescens | marcescens | UMH12 | CP018930.1 |
| Serratia marcescens | marcescens | 1274 | CP019927.2 |
| Serratia marcescens | marcescens | BWH-23 | CP020501.1 |
| Serratia marcescens | marcescens | 95 | CP020503.1 |
| Serratia marcescens | marcescens | BWH-35 | CP020507.1 |
| Serratia marcescens | marcescens | 332 | CP021164.1 |
| Serratia marcescens | marcescens | S2I7 | CP021984.1 |
| Serratia sp. | sp | MYb239 | CP023268.1 |
| Serratia marcescens | marcescens | SOLR4 | CP025698.1 |
| Serratia marcescens | marcescens | FDAARGOS_65 | CP026050.1 |
| Serratia sp. | sp | SSNIH1 | CP026383.1 |
| Serratia marcescens | marcescens | GN26 | CP026650.1 |
| Serratia marcescens | marcescens | AR_0027 | CP026702.1 |
| Serratia marcescens | marcescens | SGAir0764 | CP027300.1 |
| Serratia marcescens | marcescens | AR_0091 | CP027533.1 |
| Serratia marcescens | marcescens | AR_0099 | CP027539.1 |
| Serratia marcescens | marcescens | EL1 | CP027796.1 |
| Serratia marcescens | marcescens | KS10 | CP027798.1 |
| Serratia marcescens | marcescens | AR_0124 | CP028946.1 |
| Serratia marcescens | marcescens | AR_0130 | CP028947.1 |
| Serratia marcescens | marcescens | AR_0123 | CP028948.1 |
| Serratia marcescens | marcescens | AR_0121 | CP028949.1 |
| Serratia marcescens | marcescens | CAV1761 | CP029449.1 |
| Serratia marcescens | marcescens | AR_0131 | CP029715.1 |
| Serratia marcescens | marcescens | AR_0122 | CP029746.1 |
| Serratia marcescens | marcescens | N4-5 | CP031316.1 |
| Serratia sp. | sp | 1D1416 | CP032738.1 |
| Serratia sp. | sp | P2ACOL2 | CP033162.1 |
| Serratia sp. | sp | LS-1 | CP033504.1 |
| Serratia marcescens | marcescens | N10A28 | CP033623.1 |
| Serratia sp. | sp | FDAARGOS_506 | CP033831.1 |
| Serratia liquefaciens | liquefaciens | FG3 | CP033893.1 |
| Serratia quinivorans | quinivorans | PKL:12 | CP038467.1 |
| Serratia nematodiphila | nematodiphila | DH-S01 | CP038662.1 |
| Serratia fonticola | fonticola | MS5 | CP040182.1 |
| Serratia marcescens | marcescens | WVU-001 | CP041122.1 |
| Serratia marcescens | marcescens | WVU-002 | CP041123.1 |
| Serratia marcescens | marcescens | WVU-003 | CP041124.1 |
| Serratia marcescens | marcescens | WVU-004 | CP041125.1 |
| Serratia marcescens | marcescens | WVU-005 | CP041126.1 |
| Serratia marcescens | marcescens | WVU-006 | CP041129.1 |
| Serratia marcescens | marcescens | WVU-007 | CP041130.1 |
| Serratia marcescens | marcescens | WVU-008 | CP041131.1 |
| Serratia marcescens | marcescens | WVU-009 | CP041132.1 |
| Serratia marcescens | marcescens | WVU-010 | CP041134.1 |
| Serratia marcescens | marcescens | ATCC 13880 | CP041233.1 |
| Serratia sp. | sp | KUDC3025 | CP041764.1 |
| Serratia marcescens | marcescens | E28 | CP042512.1 |
| Serratia proteamaculans | proteamaculans | 336X | CP045913.1 |
| Serratia marcescens | marcescens | 1602 | CP047391.1 |
| Serratia sp. | sp | NGAS9 | CP047605.1 |
| Serratia marcescens | marcescens | 4201 | CP047679.1 |
| Serratia marcescens | marcescens | 3024 | CP047682.1 |
| Serratia marcescens | marcescens | 2838 | CP047685.1 |
| Serratia marcescens | marcescens | 1140 | CP047688.1 |
| Serratia marcescens | marcescens | C110 | CP047691.1 |
| Serratia liquefaciens | liquefaciens | JL02 | CP048043.1 |
| Serratia marcescens | marcescens | Db11 | HG326223.1 |
| Serratia marcescens | marcescens | SMB2099 | HG738868.1 |
| Serratia plymuthica | plymuthica | NCTC8900 | LR134151.1 |
| Serratia rubidaea | rubidaea | NCTC9419 | LR134155.1 |
| Serratia plymuthica | plymuthica | NCTC8015 | LR134478.1 |
| Serratia rubidaea | rubidaea | NCTC10036 | LR134493.1 |
| Serratia quinivorans | quinivorans | NCTC13188 | LR134494.1 |
| Serratia rubidaea | rubidaea | NCTC12971 | LR590463.1 |
| Serratia marcescens | marcescens | 4928STDY7387938 | LR607375.1 |
| Serratia plymuthica | plymuthica | NCTC12961 | LS483469.1 |
| Serratia rubidaea | rubidaea | NCTC10848 | LS483492.1 |
| Serratia marcescens | marcescens | PWN146 | LT575490.1 |
| Serratia grimesii | grimesii | BXF1 | LT883155.1 |
| Serratia ficaria | ficaria | NCTC12148 | LT906479.1 |
| Serratia sp. | sp | JKS000199 | LT907843.1 |

**Table S4: Average ANI distances of CxSm1 and CxSm2 against a selection of reference genomes (See Table S3); average values per species are shown.**

| **Species** | **Average ANI distance** |
| --- | --- |
| Ficaria | 0.110193 |
| Fonticola | 0.1877325 |
| Grimensii | 0.170926 |
| Liquefaciens | 0.1665572 |
| Marcescens | 0.05372596 |
| Nematodiphila | 0.0519203 |
| Plymuthica | 0.1539145 |
| Proteamaculans | 0.1745495 |
| Rubidaea | 0.1551814 |
| Sp. | 0.09650431 |
|  |  |

**Table S5. OTU table for 16S rRNA amplicon sequencing.**

**References for supplementary information.**

1. Mendoza-Espinoza A, Koga Y, Zavaleta AI. Amplified 16S ribosomal DNA restriction analysis for identification of Avibacterium paragallinarum. Avian Dis. 2008; 52: 54-58
2. Zhu, H., Sun, S. & Dang, H. PCR Detection of *Serratia* spp*.* Using Primers Targeting *pfs* and *luxS* Genes Involved in AI-2-Dependent Quorum Sensing. *Curr Microbiol.* 2008; 57, 326–330
3. Isoe J, Collins J, Badgandi H, Day WA, Miesfeld RL. Defects in coatomer protein I (COPI) transport cause blood feeding-induced mortality in Yellow Fever mosquitoes. Proc Natl Acad Sci USA. 2011; 108:E211–7.
4. Xia Y, Zwiebel LJ.  Identification and characterization of an odorant receptor from the West Nile virus mosquito, Culex quinquefasciatus. Insect Biochem Mol Biol. 2006; 36: 169-76.
